# Factors affecting center of pressure in older adults: the Framingham Foot Study

**DOI:** 10.1186/1757-1146-6-18

**Published:** 2013-05-08

**Authors:** Thomas J Hagedorn, Alyssa B Dufour, Yvonne M Golightly, Jody L Riskowski, Howard J Hillstrom, Virginia A Casey, Marian T Hannan

**Affiliations:** 1 Institute for Aging Research at Hebrew SeniorLife, Boston, MA, USA; 2 Department of Epidemiology, Thurston Arthritis Research Center Injury Prevention Research Center Carolina, University of North Carolina, Wilmington, USA; 3 Harvard Medical School, Boston, MA, USA; 4 Glasgow Caledonian University, Glasgow, Scotland; 5 Hospital for Special Surgery, New York, NY, USA

## Abstract

**Background:**

Although aberrant foot movement during gait has been associated with adverse outcomes in the lower extremities in clinical patients, few studies have analyzed population differences in foot function. The purpose of this study was to assess demographic differences in foot function in a large population-based study of community-dwelling adults.

**Methods:**

Participants in this study were from the Framingham Foot Study. Walking data were collected from both feet using a Tekscan Matscan pressure mat. Foot function was characterized using the center of pressure excursion index (CPEI). T-tests were used to assess differences between population subsets based on sex, and in men and women separately, age, body mass index (BMI), physical activity and in women, past high heel use.

**Results:**

There were 2111 participants included in this analysis. Significant differences in CPEI were noted by sex (p< 0.0001), by age in women (p = 0.04), and by past high heel use in women (p = 0.04).

**Conclusions:**

Foot function during gait was affected by sex, as well as by age and shoe-wear in women, but not by BMI or physical activity. Future work will evaluate possible relations between CPEI and outcomes such as falls, sarcopenia, and lower extremity function.

## Background

Foot function affects overall foot and lower extremity health. Prior research has linked abnormal foot function to adverse outcomes, such as lower extremity joint injuries [1,2] and foot deformities [3]. Although research has linked age [4,5], weight [4,6-8], shoe-wear [9] and body mass index (BMI) [7,10-12], to differences in regional foot loading patterns, the effect of these variables on foot function in a community-dwelling sample of adults is unknown. Moreover, many of these studies have small sample sizes and highly selective inclusion and exclusion criteria, which minimize the generalizability of their results. The purpose of this study was to identify factors related to foot function in a population-based cohort of community-dwelling adults.

## Methods

### Study population

Data were obtained from the Framingham Foot Study [13], a population-based cohort of ambulatory adults residing in Framingham, Massachusetts, USA. The study population was drawn from two cohorts of the Framingham Heart Study. The Framingham Original Cohort was started in 1948 to investigate risk factors of Heart disease [14]. Participants were selected through a two thirds sample of the town of Framingham, and have been examined biennially since. The Framingham Offspring Cohort is composed of adult children of the Original cohort members and their spouses who reside in or around Framingham. The Offspring cohort was started 1972 to investigate familial risk factors of heart disease, and participants have been examined every four years since [15]. The Framingham Foot Study was approved by the Hebrew SeniorLife and Boston University Medical Center’s Institutional Review Boards. Participants provided written, informed consent prior to enrolment. We conducted cross-sectional analyses using the information collected in this cohort.

Between 2002 and 2008, Framingham Foot Study participants were seen for a data collection protocol that included a walking plantar pressure assessment, a validated foot examination, assessments of clinical variables (age, weight, height), and questionnaire-based assessments of activities and shoe-wear. Analysis inclusion criteria for this study required availability of foot function (center of pressure excursion index (CPEI)) data for at least one foot and valid data on age, BMI and physical activity (the Physical Activity Scale for the Elderly or PASE) [16]. As the CPEI requires a heel-to-toe gait pattern, participants who did not have this gait pattern starting in the heel were excluded.

### Data collection

Plantar pressure data were collected from a Tekscan Matscan (Tekscan Inc, Boston) at 40 Hz. Participants walked at a self-selected pace and scans were collected using the two-step method (i.e., the foot strikes the mat on the second step) [17]. One trial was collected for each foot. During testing, participants were allowed to practice walking across the mat. Scans were repeated in instances where participants struck the mat with the wrong foot, altered their gait to strike the mat, or failed the strike the mat with their whole foot.

**Figure 1:**
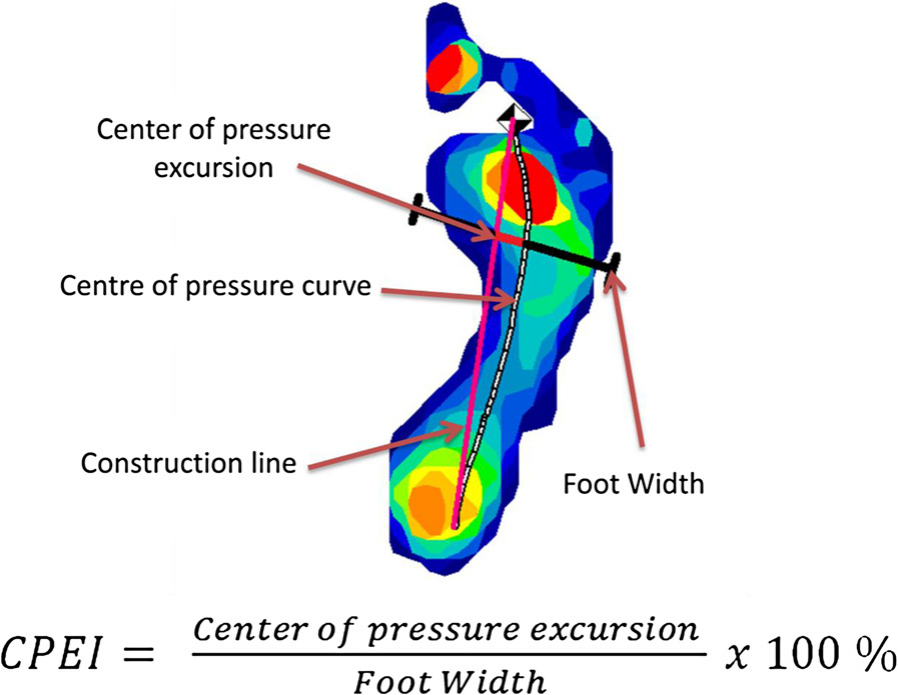
Calculation of the center of pressure excursion index (CPEI) in the Framingham Foot Study participants, 2002–08.

Foot function was characterized using the CPEI, a measure of dynamic foot function. CPEI has previously been shown to discriminate between feet clinically determined to have planus versus rectus and planus versus cavus foot types [18,19], whereas other measures of function typically only distinguish planus versus cavus feet [19]. A comparison of data from two independent raters shows that CPEI has high inter- and intra- tester reliability [18]. CPEI is calculated by drawing a line connecting the first and last point in the foot’s center of pressure, and then measuring the distance between this line and the center of pressure in the distal third tertile of foot length as a percentage of foot width (see Figure 1). A lower CPEI indicates a more pronated foot, while a higher CPEI indicates a more supinated foot during the stance phase of gait [18]. For participants that had two CPEI measures (one on each foot), only the CPEI measure that deviated the most from the median was used in the analysis. BMI was calculated from height and weight measurements collected using a calibrated stadiometer and balance beam scale. Physical activity was assessed in a subset of participants using the PASE [16] with possible scores ranging from 0 to >400; the range for participants in this study was 0 to 382. High heel use, in women only, was self-selected from a list of shoe types across three past age periods (ages 20–29, 30–44, and 45–65) in response to the question “What type of shoe did you usually wear?” [13].

### Statistical analysis

Age groups were dichotomized as ≥ 65 years or < 65 years to provide information on older adults at a common population cut-point. BMI groups were dichotomized by obesity using the cut-off ≥ 30 kg/m^2^ or < 30 kg/m^2^. PASE scores were dichotomized at the sex-specific median, with the median being 134.5 for men and 115.5 for women.

Women were grouped into those who had always worn (all three age periods), sometimes (at least one, but not all age periods), and never worn high heels (referent). Sex-specific t-tests were used to compare CPEI distributions between age, BMI, and PASE groups. A t-test was used to compare CPEI between sexes. Linear regression was used to compare high heel groups using those who had never worn high heels as the referent, and to model the relation between CPEI and continuous age, BMI, and PASE measurements. To further examine the effect of age on CPEI, we categorized age as <55 years, 55–65 years, 65–75 years and 75+ years. Linear regression was used to compare CPEI among these age groups, using those <55 years of age as the referent. Alpha was set to 0.05.

## Results

Of 2111 participants, 1154 (54.7%) were women (Table 1). The range in age was 36 to 98 years, and in BMI was 14.6 to 57 kg/m^2^. Mean CPEI was smaller (p <0.0001) among women compared to men (Table 2). Older women had smaller mean CPEI values (p = 0.04) compared to women of ages < 65 years. When analyzed in smaller age groups, both men and women over the age of 75 had significantly lower CPEI than those under 55. Mean CPEI did not differ by levels of BMI or PASE score, but was significantly lower in women who “always wore” high heeled shoes in the past (p = 0.04). Continuous models showed a significant relation between CPEI and age in both men (p = 0.0035) and women (p < 0.001), but not with BMI (p = 0.56 in men; p = 0.09 in women) or PASE score (p = 0.38 in men; p = 0.51 in women) (Table 3).

**Table 1:** Descriptive statistics (mean ± SD) for the participants in the Framingham Foot Study

	**Men**	**Women**	**Total**
N	957	1154	2111
Age	67.0±9.99	66.6±10.28	66.8±10.15
Body mass index (BMI)	29.0±4.67	27.9±5.84	28.4±5.37
Physical activity scale for the elderly (PASE)	142.0±75.17	125.6±65.08	132.9±70.20

**Table 2:** T-Tests for covariates associated with CPEI in the men and women of the Framingham Foot Study

**Men**	**N**	**Mean CPEI**	**Standard deviation**	**p**	**Women**	**N**	**Mean CPEI**	**Standard deviation**	**p**
Sex	957	16.80	9.60		Sex	1154	12.29	9.98	**<.0001**
Age < 65	418	17.37	9.60	0.12	Age < 65	551	12.93	9.96	**0.04**
Age ≥ 65	539	16.40	9.63		Age ≥ 65	603	11.71	9.98	
BMI < 30	608	17.03	9.67	0.36	BMI < 30	805	12.10	10.10	0.31
BMI ≥ 30	349	16.45	9.55		BMI ≥ 30	349	12.75	9.70	
PASE <134.5	283	15.84	9.45	0.63	PASE <115.5	353	11.74	9.62	0.59
PASE ≥134.5	283	16.23	9.57		PASE ≥115.5	353	11.35	9.63	
					Never wore High heeled shoes(ref)	496	13.07	9.76	-
					Sometimes wore high heeled shoes	471	11.85	10.21	0.0586
					Always wore High heeled shoes	187	11.35	9.87	**0.0448**

**Table 3:** Mean center of pressure excursion index across age groups in the men and women of the Framingham Foot Study

**Men**	**N**	**Mean CPEI**	**P**	**Women**	**N**	**Mean CPEI**	**P**
Age <55	91	18.53 ± 9.94	-	Age < 55	125	14.07 ± 10.39	-
55-65	327	17.04 ± 9.49	0.191	55-65	426	12.59 ± 9.81	0.145
65-75	314	16.73 ± 9.66	0.115	65-75	336	12.58 ± 10.06	0.155
75+	225	15.94 ± 9.6	0.03	75+	267	10.62 ± 9.78	0.001

## Discussion

The purpose of this study was to evaluate demographic factors associated with foot function in a community-dwelling, population-based large sample of men and women. We found significant differences in CPEI in groups stratified by age, sex, and past high heel use, but not by BMI or physical activity. Continuous models showed similar results in both men and women. These results indicate that future studies of foot function should consider the effects of sex, age, and history of high heel use as these factors may affect an outcome of interest.

Similar to prior studies [20,21], this study found foot function differs by sex. This work noted that women displayed a lower CPEI. Smaller CPEI values are associated with greater amounts of pronation [18,19]. This in part may be due to women having a more planus foot structure then men. Recent literature also suggests that arch height is reduced post-partum [22]. As over-pronation has been clinically observed to be associated with several pedal pathologies (hallux valgus, hallux limitus, hallux rigidus, posterior tibial dysfunction, etc.) [23], this may mean women are at a greater risk of foot issues compared to men. The higher CPEI seen in men is consistent with prior work showing higher forces in the lateral metatarsals in men, which may correspond with a more supinated foot position [21]. Thus, the current work extends the information available in the medical literature.

In this study, age older than 65 years was associated with lower CPEI in women, indicating more pronated foot function during gait. A similar magnitude of effect was noted in men, but was only significant in the continuous models. Clinicians have qualitatively observed that arches may become lower as persons age, and this observation is consistent with studies noting increased rates of flat feet [24] and pronated feet [5] with increasing age. If older individuals are becoming more planus they may be predisposed to a greater incidence of associated pathologies. As foot pathologies have been linked to functional limitations [25] and fall risk [26], this age related change might have significant consequences over time While there was no statistically significant difference in mean foot function between men ≥ 65 or < 65 years, both sexes had significantly lower CPEI among those 75 years or older, relative to those under 55, with a trend towards decrease over time in the other age groups. Future research should more thoroughly investigate biomechanical changes in the foot with age , as well as sex differences with age in foot function as perhaps it may help explain differences in rates of knee injury [27] and joint degeneration [28] between the sexes.

Individuals with higher BMI are suspected of having a higher prevalence of flat feet [24], which may be associated with increased foot pronation during the stance phase of gait [23]. However, it is unclear whether static measures are accurate predictors of foot function [29-31], and few studies have directly assessed the relation between BMI and foot function. Previous research in small groups of adult volunteers has found that obese participants had larger plantar contact areas [32] and higher pressure under the forefoot during stance [11]. Messier et al. [33] found obese participants had significantly greater rearfoot eversion relative to normal weight participants in a 2D motion capture analysis of female volunteers. In our current analysis, CPEI was unaffected by BMI, suggesting no relation between foot function and obesity. Several factors may account for the difference between this result and previous research. As CPEI measures foot function using the distribution of load under the foot over time [18], it may not be directly comparable to previous studies of static plantar pressure [10] and kinematics [33]. Moreover, this study was population-based, while previous studies used small samples of volunteers [11,32,33]. Further, our study population was older (with an age range of 36 to 98 years versus samples primarily in their twenties [11] and forties [32]) and had a lower mean BMI (mean BMI was 28.4 kg/m^2^ versus 41.1 kg/m^2^ for obese group in the Messier study [33]).

Given the link between foot deformities and muscle weakness in diabetic patients [34] and fallers [35], and between foot deformities and altered foot biomechanics [23], physical activity may affect foot function. In the current study, however, CPEI was not affected by physical activity levels in either continuous or categorical analyses. While PASE does not measure physical function directly, it has been shown to be associated with a number of physiological measures of physical function [16]. This result provides preliminary evidence that foot function as measured in the current study may not be significantly related to physical activity.

Both finite element modeling [36] and a study of young volunteers in Taiwan [37] found that high heels increased medial forefoot and toe loading in shod feet. However, the effects of habitually wearing high heels on barefoot gait are not well understood. In this study, women who always wore high heels over their adult lifespan had significantly lower CPEI than those who never wore them. Lower CPEI is consistent with the higher medial forefoot loading observed previously by these authors, and thus may indicate that changes from high heel use have a modifying effect on plantar loading. These results are in agreement with work in children showing that past shoe use can affect foot structure [9,38]. Future research should look at the specific effects of past shoe-wear on plantar loading in both older women and men.

This study has several strengths and limitations worth noting. Because the study design was cross-sectional, causal relations between foot function and the factors under study cannot be inferred. Due to examination time constraints, only one plantar pressure scan per foot was obtained from each participant and thus, there is likely a larger degree of measurement error. This limitation is mitigated by the large sample size of the study, but if this error had an effect on the results, it would act to bias towards a null effect between variables rather than create a false positive [39]. There were also several strengths to this population-based study. The study had a large sample size spanning a wide age range (36 to 98 years) and body size (BMI ranged 14.6 to 57 kg/m^2^), in addition to including both men and women.

## Conclusions

This study showed that there are sex, age, and high heel use-related differences in foot function (as measured by CPEI) in a large population-based sample of men and women. These results should be helpful in informing future research and analysis of foot biomechanics. Future work will evaluate the relation between CPEI and outcomes such as falls, as well as lower extremity function, injury, musculoskeletal disorders, and disease.

## Competing interests

The authors have no competing interests to report.

## Authors’ contributions

TJH contributed to the analysis and interpretation of data and drafted the original manuscript. ABD carried out the statistical analyses, contributed to the interpretation of data and the revision of the manuscript. YMG made substantial contributions to the drafting and revision of the manuscript. JLR participated in the interpretation of data and the drafting and revision of the manuscript. HJH participated in the study conception and design and provided critical revision of the manuscript for intellectual content. VAC made substantial contributions to the drafting and revision of the manuscript. MTH conceived of the study, was responsible for the acquisition of data, contributed to the analysis and interpretation of data, and provided critical revision of the manuscript for intellectual content. All authors read and approved the final manuscript.
